# Gender Differences in Smoking Initiation and Cessation Associated with the Intergenerational Transfer of Smoking across Three Generations: The Nagahama Study

**DOI:** 10.3390/ijerph19031511

**Published:** 2022-01-28

**Authors:** Sachiko Nakagawa, Yoshimitsu Takahashi, Takeo Nakayama, Shigeo Muro, Michiaki Mishima, Akihiro Sekine, Yasuharu Tabara, Fumihiko Matsuda, Shinji Kosugi

**Affiliations:** 1 Department of Medical Ethics and Medical Genetics, Kyoto University School of Public Health, Kyoto 606-8501, Japan; nakagawa@jpma.or.jp; 2 Department of Health Informatics, Kyoto University School of Public Health, Kyoto 606-8501, Japan; takahashi.yoshimitsu.3m@kyoto-u.ac.jp (Y.T.); nakayama.takeo.4a@kyoto-u.ac.jp (T.N.); 3 Department of Respiratory Medicine, Nara Medical University, Nara 634-8521, Japan; smuro@naramed-u.ac.jp; 4 Department of Respiratory Medicine, Kyoto University Graduate School of Medicine, Kyoto 606-8501, Japan; mishima@kuhp.kyoto-u.ac.jp; 5 Center for Preventive Medical Science, Chiba University, Chiba 260-8670, Japan; sekine.akihiro@chiba-u.jp; 6 Center for Genomic Medicine, Kyoto University Graduate School of Medicine, Kyoto 606-8501, Japan; tabara@genome.med.kyoto-u.ac.jp (Y.T.); fumi@genome.med.kyoto-u.ac.jp (F.M.)

**Keywords:** smoking cessation, smoking initiation, gender difference, vulnerable population, family history, intergenerational relations, logistic regression analysis, cross-sectional study, WHO, FCTC, MPOWER

## Abstract

Global tobacco policies lowered overall and male smoking rates, but female smoking rates have remained unchanged. Parent–child studies revealed the effects of parental smoking, but gender differences had mixed results. We investigated the effects of long-term smoking behavior in families over three generations in order to clarify gender differences. A cross-sectional study in a community-based genome cohort was conducted using a self-reported questionnaire. A total of 8652 respondents were stratified by gender regarding smoking initiation. A logistic regression analysis was performed to analyze the family smoking history. A total of 2987 current smokers and ever-smokers were compared regarding smoking cessation. With respect to smoking initiation, women were affected by their smoking mothers (odds ratio (OR), 2.4; 95% confidence interval (CI), 1.8–3.2) and grandmothers (OR, 1.7; CI, 1.1–2.4). Women who continued smoking were affected only by their smoking mothers (OR, 1.6; CI, 1.05–2.49). In conclusion, gender differences in smoking initiation and cessation are possibly associated with family smoking history. Mothers and grandmothers were shown to have a strong influence on women with respect to both smoking initiation and cessation. Future research should focus on providing evidence for effective gender-specific intervention programs to curb long-term smoking in women.

## 1. Introduction

In the last 50 years, evidence-based tobacco control policies have contributed to the worldwide decrease in the prevalence of cigarette smoking among adults [1]. Trends in many countries over the past 25 years have shown that smoking rates for men have dropped significantly more than those for women [2]. The largest imbalance has been found in the southeast Asia and western Pacific regions [3]. Japan, which is located in the aforementioned regions, has ratified the WHO Framework Convention on Tobacco Control (WHO FCTC). In 2008, WHO introduced the “MPOWER” measures to assist in the country-level implementation of effective interventions to reduce the demand for tobacco, contained in the WHO FCTC: monitor tobacco use and prevention policies (M); protect people from exposure to second-hand tobacco smoke (P); offer help to quit tobacco use (O); warn about the dangers of tobacco (W); enforce bans on tobacco advertising, promotion, and sponsorship (E); raise taxes on tobacco (R). In an attempt to further lower smoking rates, smoking cessation medication began being covered by health insurance policies in 2006, and tobacco tax increases and restrictions on smoking areas started being promoted. However, MPOWER measures have been found to be insufficient [4]. Both the smoking rate (29.4% of men and 7.2% of women smoked daily in 2019) [5] and the decline in smoking rate show significant gender differences. In order to reduce the smoking rate, smoking cessation policies and the prevention of smoking initiation are both important factors. Regarding gender differences in smoking cessation, women are more likely to have difficulty maintaining long-term abstinence from smoking than men including those with even moderate addiction [6], those between the ages of 30 and 55 (approximately) [6], those with some smoking cessation therapy [7,8], and those who consume alcohol [9]. Some arguments have been put forward stating that the gender differences observed in clinical trials [10] cannot be translated into real-world settings [11], but a recent study on the utilization of National Tobacco Cessation Services among female smokers also suggested that further research considering gender-specific tobacco cessation support was needed [12].

A WHO survey from 2015 on smoking initiation among women and girls noted that a better understanding of the key factors affecting this situation was needed in order to address the growing global pandemic of female smokers and the looming impact on women’s health [13]. The most-studied environmental influences thus far include parental, sibling, and peer relationships. Parental smoking has been shown to strongly and significantly affect smoking initiation among offspring [14]. Compared to offspring of nonsmokers, those of persistent smokers had twofold smoking rates (OR, 1.96; CI, 1.31–2.93). Maternal smoking was more strongly associated with youths’ regular smoking than parental smoking (ORs, 3.12; CI, 1.58–6.16 vs. 1.47; 0.87–2.49) [15]. The effect of parental smoking appears to vary on the basis of the parent’s sex/gender, which is likely to be related to the traditional roles of mothers and fathers [15]. Regarding the influence of smoking grandparents, results indicate direct linkages between both grandparent and parent smoking (OR, 4.53; CI, 2.57–7.97) and parent and offspring smoking (OR, 2.91; CI, 1.60–5.31); however, the direct link between grandparents and grandchildren was not significant (OR, 2.25; CI, 0.96–5.23, *p* < 0.10) [16]. According to a study in Finland, both girls and boys had higher odds for daily smoking, if any of the four grandparents were current smokers. Results indicated the significant odds between paternal grandfather and girls (OR, 2.63; CI, 1.59–4.33), paternal grandmother and girls (OR, 1.71; CI, 1.01–2.90), paternal grandmother and boys (OR, 3.02 (1.50–6.09), maternal grandfather and boys (OR, 1.99; CI, 1.02–3.89), maternal grandmother and girls (OR, 2.88; CI 1.88–4.36), and maternal grandmother and boys (OR, 2.03; CI, 1.00–4.10). However, when parents’ and grandparents’ smoking status was included in the same model, grandparent smoking generally lost statistical significance [17]. Despite the potential effects of smoking grandparents, the results were inconsistent. Furthermore, this has not been studied in the Asia Pacific region, which has a particularly large gender difference with respect to smoking rate.

We hypothesized that the integrated analysis of smoking behavior over three generations would reveal how it may consistently affect the next generation, which is an extension of previously reported parent–child studies that only investigated parental influences. To compare smoking behavior with that of parents and grandparents to investigate whether there are any gender differences between smoking initiation and cessation, new evidence-based, gender-specific tobacco policies can be implemented with the aim of reducing the smoking rate among women. Therefore, it is necessary to clarify the gender differences in smoking behavior across generations. The primary aim of this work was to perform a cross-sectional study to investigate how gender differences affect smoking initiation and cessation in a Japanese genome cohort, focusing on intergenerational transmission over three generations, from grandparents to their children and, subsequently, from their children to their grandchildren.

## 2. Materials and Methods

### 2.1. Study Design and Participants

**Figure 1:**
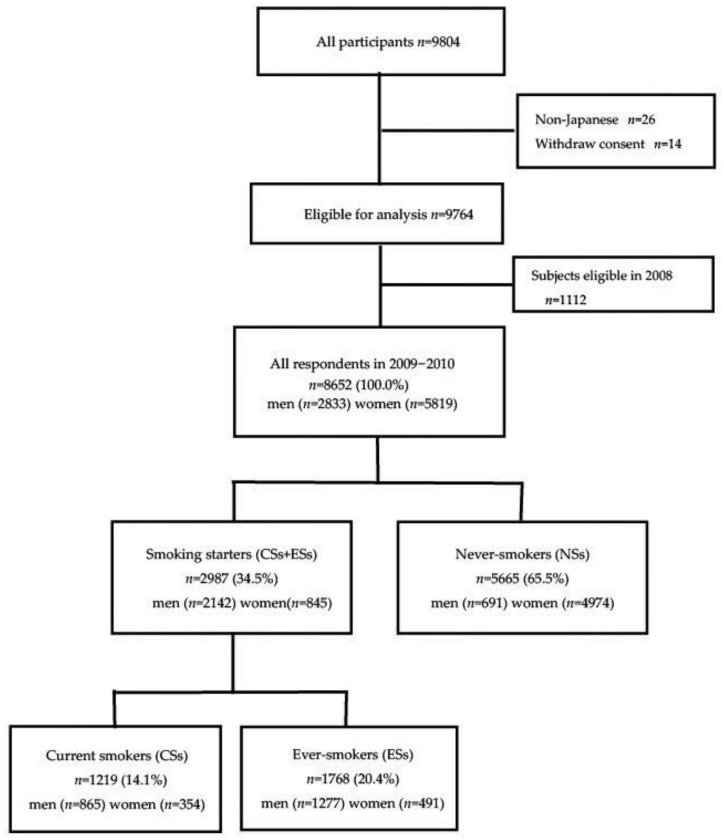
Participant flow diagram.

The present study was a cross-sectional study using the Nagahama Prospective Cohort for Comprehensive Human Bioscience (The Nagahama Study). The cohort was recruited from the general population of Nagahama city, Japan, which has a population of 125,000. A total of 9804 participants were recruited via mass communication by the local government and citizen organizations from 2008 to 2010. Of these participants, we excluded 26 non-Japanese participants using PCA (polymerase cycling assembly) [18] and 14 participants withdrew their consent, which left a total of 9764 participants for the analysis. A structured questionnaire on smoking was implemented from 2009 to 2010 for baseline investigation, and 8652 subjects were registered from 2009 to 2010 for our analyses. Figure 1 shows the study flowchart. A copy of the questionnaire used to capture the data is provided in the Supplementary Materials.

The researchers and city employees held an information session for the residents regarding the present study. Japan has universal health coverage, and this study included the Specific Health Examination mandated by Japanese national laws for medical insurers to prevent against noncommunicable diseases [19]. The cohort was primarily aimed at healthy subjects, and participants had to meet the following criteria: (1) be aged between 30 and 74 years old, (2) be able to participate in health examinations independently, (3) have no difficulty in communication, (4) have little serious diseases/symptoms or health issues, and (5) have voluntarily decided to participate in the study. The municipal government staff made appointments for health examinations by telephone, and the participants were registered at the site of the health examination. Written informed consent was obtained from all of the participants prior to their health examination. The response rate was 100% and the interviews were carried out at the location of the medical checkup. All protocols and informed consent procedures were approved by the Kyoto University Graduate School and Faculty of Medicine Ethics Committee, the Ad Hoc Review Board of the Nagahama Cohort Project, and the Nagahama Municipal Review Board of Personal Information Protection. This study was carried out in accordance with approved guidelines. Smoking status, intergenerational variables, demographic and environmental variables, and variables of noncommunicable diseases were collected using an anonymous self-administered postal questionnaire.

### 2.2. Outcome Variables

#### Assessment of Smoking Status

Smoking status was categorized into three clinical groups: current smokers (CSs), ever-smokers (ESs), and never-smokers (NSs). These groups were respectively defined as those who smoke every day or some days, those who have quit smoking, and those who have no experience of smoking. To assess the factors of smoking initiation, smoking starters (CSs and ESs) were compared with never-smokers (NSs). To assess the factors of successful cessation, CSs were compared with ESs.

### 2.3. Correlated Variables

#### 2.3.1. Intergenerational Variables

The main correlated variable was the intergenerational transmission of smoking across three generations. The intergenerational variables consisted of dichotomous indicator variables, i.e., whether or not the mother, father, grandmother, grandfather, or siblings smoke. All answers were chosen as either yes or no (binary scoring). When the mother was recorded as a smoker, we also determined her smoking status during pregnancy (1 = yes; 0 = no; unknown = excluded).

#### 2.3.2. Years of Smoking and Smoking Initiation

The number of years that CSs and ESs had been smoking for was determined. Both their age and the number of cigarettes smoked per day (CPD) at the time of smoking initiation were also provided as the smoking initiation status. Age was categorized as either <20 or ≥20 years, and CPD was categorized as 1–10, 11–20, or ≥21.

#### 2.3.3. Variables of Alcohol Consumption

As alcohol consumption could have been a confounder, the variable of alcohol consumption consisted of four indicator variables according to the answer to the following question: “How much do you drink a day, in terms of glasses of refined Japanese Sake?” A glass (180 mL) of refined Sake (rice wine) is equivalent to a medium bottle (500 mL) of beer, 110 mL of Shochu (alcohol content 25%), a glass (double, 60 mL) of whisky, and two glasses (240 mL) of wine.

#### 2.3.4. Demographic and Environmental Variables

Age and spousal smoking status were evaluated as moderators of the association between gender and smoking behavior, smoking initiation, and smoking cessation. Spousal smoking was indicated as either yes or no (binary scoring). Body mass index (BMI) was evaluated as a moderator of the association with smoking cessation. Participants were grouped into the following categories according to BMI: <25.0 and ≥25.0.

#### 2.3.5. Variables of Noncommunicable Disease

It is known that smoking facilitates the development of diabetes mellitus by affecting the function of insulin in lowering blood sugar levels, triggering dyslipidemia by hindering lipid metabolism, and increasing blood pressure due to the action of constricting blood vessels. In the Specific Health Examination and Specific Health Guidance, smoking habits are counted as an additional risk when combined with high blood pressure, dyslipidemia, or high blood sugar, in addition to abdominal obesity. There are three types of blood tests used in the Specific Health Examination: lipid, liver function, and blood sugar. We also assessed renal function. Glutamic oxaloacetic transaminase (GOT), glutamic pyruvic transaminase (GPT), gamma-glutamyl transferase (γ-GTP), total cholesterol (Tch), blood sugar (BS), hemoglobin A1c (HbA1c), systolic blood pressure (BP), and serum creatinine (SerumCr) were evaluated as variables of noncommunicable disease.

### 2.4. Statistical Analysis

Using data from all of the participants, we examined gender differences in smoking initiation and cessation. The participants were stratified by gender. We compared each variable between the two groups using Fisher’s exact test and *t*-test. We also performed an analysis of variance among the three groups. In order to identify the potential association between the independent variables and smoking behavior, all variables were entered into univariate logistic regression models. Multiple logistic regression analysis was performed to identify the correlated factors of smoking behavior. When a high degree of correlation existed between variables, they were not entered together in the same multivariable model. Multicollinearity was assessed using correlation coefficients and the variance inflation factor (VIF). Given the known relationship between smoking cessation and menstruation status [20], age could have affected gender differences in the success rate of smoking cessation. Age was, thus, categorized into groups (i.e., 30–55 and ≥56 years of age). Multivariate logistic regression models were prepared to estimate the factors correlated with smoking initiation and cessation. Those variables included in the adjusted models were based on the existing knowledge of risk factors for smoking behavior. Continuous variables were categorized for regression analyses. Multivariate logistic regressions were subsequently used to extend the analysis and to assess the role of correlated variables when entered into the equation simultaneously. The covariates for the adjusted models were age group, spousal smoking status, and family smoking history. We evaluated the interaction between age and years of smoking in the final models using main-effect terms. Sensitivity analyses were performed to test the influence of the number of years of smoking. To assess the influence of smoking during pregnancy, we calculated the odds ratio using Fisher’s exact test. All statistical analyses were performed using Software R version 4.1.2 (http://www.r-project.org/, accessed on 14 January 2022). A *p*-value <0.05 was considered statistically significant.

## 3. Results

Among the 8652 respondents who met the inclusion criteria, 2833 were men and 5819 were women. Overall, 14.1% (*n* = 1219) were CSs, 20.4% (*n* = 1768) were ESs, and 65.5% (*n* = 5665) were NSs. The rate of smoking among men (30.5%, 865/2833) was higher than that among women (6.1%, 354/5819). More women were NSs, with a rate of 85.5% compared with a rate of 24.4% among men. Table 1 shows a comparison of the demographic background and indices of noncommunicable diseases among the three groups.

### 3.1. Gender Differences in Smoking Initiation

The results of the logistic regression analysis are shown in Table 2. Gender differences were observed to significantly correlate with smoking initiation in the final model. Women with smoking mothers (odds ratio (OR), 2.4; 95% confidence interval (CI), 1.8–3.2) and grandmothers (OR, 1.7; 95% CI, 1.1–2.4) had a higher likelihood of being a CS or an ES. Men were not affected by their smoking mothers or grandparents, while women were more likely to be affected by their smoking mothers and grandmothers. Age showed opposite trends with respect to gender. Women aged ≤55 years had significantly more smoking experience than those aged ≥56 years (OR, 3.6; 95% CI, 3.0–4.3). On the contrary, men aged ≤55 years had less smoking experience than those aged ≥56 years (OR, 0.6; 95% CI, 0.5–0.7). Both men and women were affected by their fathers (OR, 1.4; 95% CI, 1.2–1.7 and OR, 1.3; 95% CI, 1.1–1.5, respectively) and siblings (OR, 1.8; 95% CI, 1.4–2.2 and OR, 2.3; 95% CI, 1.9–2.7, respectively).

### 3.2. Gender Differences in Smoking Cessation

The variables used for the additional adjustment for smoking cessation were BMI (<25.0 or ≥25.0), CPD, age at the time of smoking initiation, spousal smoking status, and alcohol consumption. As shown in Table 3, gender differences were observed to be significantly correlated with smoking cessation in the final model. Women who continued smoking were affected by their smoking mothers (OR, 1.6; 95% CI, 1.0–2.4), which is consistent with the findings of smoking initiation. Conversely, men with smoking fathers (OR, 1.8; 95% CI, 1.4–2.1), grandfathers (OR, 1.5; 95% CI, 1.1–2.0), spouses (OR, 1.5; 95% CI, 1.1–2.1), and siblings (OR, 1.3; 95% CI, 1.0 –1.6) continued smoking. Men aged ≤55 years of age continued smoking compared with those aged ≥56 years (OR, 2.4: 95% CI, 2.0–2.9), whereas no significant differences were found in women. In men, obesity was defined as having a BMI of more than 25.0. Obesity in CSs was observed significantly less than in ESs (OR, 0.8; 95% CI, 0.6–0.9). The results of the sensitivity analyses revealed that when years of smoking were entered in the model, the age of women showed a reverse effect, while the age of men did not have any influence. Age was, thus, retained in the model.

### 3.3. Association between Maternal Smoking during Pregnancy and Smoking Initiation

Given the correlation coefficient of 0.60 (*p* < 0.01) between smoking mothers and antenatal maternal smoking, maternal smoking during pregnancy was excluded to avoid multicollinearity in the multivariate analysis. Fisher’s exact test showed gender differences in maternal smoking during pregnancy. The odds ratios were 0.28 (smoking/nonsmoking mother in pregnancy = 17/60) for CSs and ESs and 0.04 (1/23) for NSs among men, and 0.25 (15/60) for CSs and ESs and 0.36 (27/76) for NSs among women. Antenatal maternal smoking affected smoking initiation among men, but not significantly (OR, 6.4; *p* = 0.06; 95% CI, 0.9–283.6). On the contrary, it did not affect women (OR, 0.7; *p* = 0.37; 95% CI, 0.3–1.5).

## 4. Discussion

This cross-sectional study in a Japanese genome cohort, based on data from the general population of Nagahama city in Japan, demonstrated that gender differences were associated with the intergenerational transmission of smoking behavior across three generations. There were three key findings in this study. The first, with respect to smoking initiation, was that women were affected by their mothers and grandmothers. The second, with respect to smoking cessation, was that smoking in all family members except the mother and the grandmother affected men, whereas women were only affected by their smoking mothers. The third, with respect to age, was that, in the case of men, the smoking initiation rate was significantly lower in the younger age group and the smoking rate was significantly lower in the older age group; however, in women, the smoking initiation rate was significantly higher in the younger age group and the smoking rate was not different between younger and older age groups. Under the same tobacco control for both men and women, there was no significant impact on curbing smoking rates, because female smokers were in the minority. Given the fact that the smoking rate in younger women has not declined, as shown in this study, the WHO report in 2019 showed that the smoking rate among women in high-income countries was still the highest among all countries (16.4%), more than four times the average rate seen in low- and middle-income countries (3.5%). In contrast, the highest rate among men was seen in middle-income countries (35.3%), where it was almost double the average rate found in low-income countries (20.2%). Among women in high-income countries, it is globally rising [21]; thus, the use of the same tobacco control methods for men and women may not sufficiently restrain the smoking behavior of women in their social advancement.

The effects of parental smoking differed by gender for both smoking behavior and nicotine dependence [22]. In the present study, the smoking status of mothers significantly increased the likelihood of smoking initiation among women but not among men, which is consistent with a previous report [15,23]. Moreover, we found that grandmothers had a stronger influence on smoking initiation among women compared with fathers and grandfathers. In contrast to our results, a previous study indicated that the direct linkages between grandparents and grandchildren were not significant [16,17]. However, these studies also suggested that their offspring represented the combined influence of the family social environment, as well as shared genetic factors affecting smoking initiation and nicotine addiction.

Gender differences may reflect the distinctive sexual dimorphism of the brain, including hormonal and structural factors that emerge during fetal development [24]. Similarities in smoking behavior could be due to shared genes, particularly those involved in nicotine metabolism (e.g., CYP2A6) or those related to the availability of brain neurotransmitters, such as dopamine and serotonin (e.g., DRD2 and 5HTTLRP) [25]. Pharmacogenetic studies have suggested associations between treatment outcomes and genes coding for drug-metabolizing enzymes (e.g., CYP2A6 and CYP2B6) [26,27,28]. CYP2A6 metabolizes nicotine. The rate of inactivation and the clearance of nicotine can ultimately influence smoking behavior, as well as the ability of smokers to quit smoking. In a recent study, women were associated with higher CYP2A6 mRNA expression, relative to men in univariate analyses with similar trends for protein levels and enzyme activity, but not significantly [29]. If a gender-specific genetic etiology of smoking cessation is found, not only is effective smoking cessation treatment possible, but tobacco control can also be implemented according to the evidence.

The WHO has stated that smoking cessation interventions should be effective for and available to vulnerable groups of people [30]. This indicates the need to consider appropriate tobacco control measures for women. According to a previous Canadian study on tobacco control, price and taxation were the only policies related to smoking behavior in both men and women, whereas the association between health education and smoking behavior was gender-specific [31]. Per capita health education expenditures were positively associated with the odds of being a nonsmoker for men (OR, 1.36; CI, 1.09–1.70) but not women (OR, 1.13; CI, 0.91–1.39) [31]. Even at a younger age, there was no evidence that school-based smoking prevention programs have a significant effect on preventing adolescent girls from smoking (overall pooled effect relative risk, 0.96; CI, 0.86–1.08) [32]. A Cochrane review for family-based programs for preventing smoking by children and adolescents regardless of gender showed moderate-quality evidence of family-based interventions having a positive impact on preventing smoking when compared to a no-intervention control (risk ratio, 0.76; CI, 0.68–0.84) [33]. However, mothers and grandmothers are often positive role models for girls, and smoking behavior is inherited, as shown in our research. This relationship could hinder the success of family-based programs. We also paid attention to the intergenerational transmission of implicit attitudes in adolescents’ smoking initiation. Mothers who had more positive implicit attitudes had children with more positive implicit attitudes. These positive implicit attitudes of adolescents predicted their smoking initiation 18 months later [34].

Gender differences in smoking rates and the declining smoking rates in Japan could be explained by the following: as smoking rates among fathers and grandfathers have declined over the last 50 years, smoking in their affected sons has also declined; meanwhile, smoking rates among women have been maintained across generations of mothers and grandmothers, resulting in no decrease in smoking initiation among women, indicating that mothers and grandmothers have a strong influence on female offspring in terms of both smoking initiation and cessation. Effective smoking prevention education tools and timing for girls should be the focus rather than smoking cessation after initiation. In order for tobacco public policy to succeed completely, it is necessary to clarify the cause of intergenerational transfer for smoking behavior, and further research is required for related evidence.

Age groups showed gender differences in smoking cessation. In previous studies, gender differences were observed in smoking cessation among respondents between 30 and 55 years of age, with women having greater difficulty abstaining from smoking [6]. One of the big gender differences is presumed to be menopause. Since dramatic changes in the hormonal environment are associated with various health problems, investigating the different effects of female hormones is a worthwhile avenue for future research.

The present study had several limitations. This was a cross-sectional study; thus, predictors could not be determined. The data were based on self-reports, allowing recall bias of the participants, especially with regard to smokers indicating their status as nonsmokers. Conversely, CSs and ESs reflect their perceptions of their parents and grandparents smoking, which can be considered even more important than the actual grandparents’ smoking status. Our setting was Nagahama City, which is a relatively populous local area in Japan. The population ratio of males to females is 48.9% to 51.2% (48.6% men and 51.4% women in Japan), along with 13.6% for ages from 0 to 14 years old, 58.2% for ages 15 to 64, and 28.2% for ages over 65 (12.1%, 59.5%, and 28.4% in Japan, respectively). While we consider our study cohort representative of the Japanese population, the small population may prevent the generalization of this study. The intensity of smoking was also self-reported and not determined. Regarding smoking cessation, one of the reasons for failure to quit smoking is nicotine addiction. We captured CPD data, but we did not determine the degree of nicotine addiction and smoking cessation therapy. We analyzed the secondary data from the Nagahama Study. We surveyed around 3000 male cases in line with a previous study [17] with 3535 samples (response rate of 37%). Even though our questionnaire response rate was 100%, secondary data processing led to a sample size of 2833 men and 5819 women. Given the small sample size, the statistical power may have been low.

## 5. Conclusions

In conclusion, we found that gender differences in smoking initiation and cessation might be attributed to differences in the influencers of smoking among family members. Compared with men, women with smoking mothers and grandmothers were more likely to be smokers, and women with smoking mothers were less likely to achieve smoking cessation. Our findings suggest that smoking mothers may continue to affect the smoking behavior of their daughters and even granddaughters. Future research should focus on whether the long-term smoking behavior of women is due to genetic factors in matrilineal families or provide evidence to help develop effective gender-specific intervention programs to curb the long-term smoking behavior of women.

## Figures and Tables

**Table 1:** Demographic background and indices of noncommunicable diseases.

**Men**	**Current** **Smokers**	**Ever-Smokers**	**Never-Smokers**
*n* (%)	865 (30.5%)	1277 (45.1%)	691 (24.4%)
Age at enrolment (years)	55 (39–63)	63 (54–68)	59 (41–67)
BMI (kg/m^2^)	22.9 (21.0–25.0)	23.5 (21.7–25.3)	23.1 (21.2–25.1)
GOT (IU/L)	23 (19–28)	24 (21–29)	23 (20–28)
GPT (IU/L)	21 (16–31)	22 (17–31)	22 (17–30)
γ-GTP (IU/L)	37 (25–60)	34 (24–57)	30 (21–46)
Tch (mg/dL)	199 (178–222)	200 (179–222)	198 (178–223)
BS (mg/dL)	89 (84–95)	91 (86–98)	89 (84–96)
HbA1c (%)	5.0 (4.8–5.3)	5.1 (4.9–5.4)	5.0 (4.9–5.3)
BP (mmHg)	125 (117–136)	128 (119–138)	126 (116–137)
Serum Cr (mg/dL)	0.8 (0.7–0.9)	0.8 (0.8–0.9)	0.9 (0.8–0.9)
**Women**	**Current Smokers**	**Ever-Smokers**	**Never-Smokers**
*n* (%)	354 (6.1%)	491 (8.4%)	4974 (85.5%)
Age at enrolment (years)	42 (36–55)	40 (34–54)	57 (44–64)
BMI (kg/m^2^)	20.3 (18.8–22.7)	20.7 (19.2–22.6)	21.5 (19.7–23.7)
GOT (IU/L)	19 (16–22)	19 (16–23)	21 (18–25)
GPT (IU/L)	14 (11–18)	14 (11–19)	16 (13–21)
γ-GTP (IU/L)	18 (14–26)	16 (13–23)	17 (14–25)
Tch (mg/dL)	194 (172–220)	197 (174–220)	211 (188–234)
BS (mg/dL)	85 (80–90)	85 (81–91)	87 (83–92)
HbA1c (%)	4.9 (4.8–5.2)	4.9 (4.8–5.2)	5.1 (4.9–5.3)
BP (mmHg)	110 (103–122)	110 (103–121)	119 (108–130)
Serum Cr (mg/dL)	0.6 (0.5–0.7)	0.6 (0.5–0.7)	0.6 (0.6–0.7)

Data are presented as the median (IQR). BMI: body mass index, GOT: glutamic oxaloacetic transaminase, GPT: glutamic pyruvic transaminase, γ-GTP: gamma-glutamyl transpeptidase, Tch: total cholesterol, BS: blood sugar, HbA1c: hemoglobin A1c, BP: systolic blood pressure, Serum Cr: serum creatinine.

**Table 2:** Gender differences in smoking initiation.

**Men**							
**Variables**		***n***	**Current and Ever-Smokers ** ***n*, %**		**Crude OR** **(95% CI)** **(*n* = 2833)**	**Adjusted OR** **(95% CI)** **(*n* = 2827)**	***p*-Value**
Age (years)	30–55	1093	779	71.3%	0.7 (0.6–0.8)	0.6 (0.5–0.7)	<0.001
	56≤	1740	1363	78.3%	ref.	ref	
Father smokes	Yes	1675	1311	78.3%	1.4 (1.2–1.7)	1.4 (1.2–1.7)	<0.001
	No	1158	831	71.8%	ref.	ref.	
Mother smokes	Yes	172	131	76.2%	1.0 (0.7–1.5)	0.9 (0.6–1.4)	0.66
	No	2661	2011	75.6%	ref.	ref.	
Grandfather smokes	Yes	308	240	77.9%	1.2 (0.9–1.5)	1.1 (0.8–1.5)	0.50
	No	2525	1902	75.3%	ref.	ref.	
Grandmother smokes	Yes	71	57	80.3%	1.3 (0.8–2.5)	1.2 (0.7–2.2)	0.62
	No	2762	2085	75.5%	ref.	ref.	
Sibling smokes	Yes	613	511	83.4%	1.8 (1.4–2.3)	1.8 (1.4–2.2)	<0.001
	No	2220	1631	73.5%	ref.	ref.	
**Women**							
**Variables**		***n***	**Current and Ever-Smokers** ***n*, %**		**Crude OR** **(95% CI)** **(*n* = 5819)**	**Adjusted OR** **(95% CI)** **(*n* = 5813)**	***p-*** **V** **alue**
Age (years)	30–55	2895	651	22.5%	4.1 (3.5–4.8)	3.6 (3.0–4.3)	<0.001
	56≤	2924	194	6.6%	ref.	ref.	
Father smokes	Yes	3065	569	18.6%	2.0 (1.8–2.4)	1.3 (1.1–1.5)	0.01
	No	2754	276	10.0%	ref.	ref.	
Mother smokes	Yes	287	102	35.5%	3.6 (2.7–4.6)	2.4 (1.8–3.2)	<0.001
	No	5532	743	13.4%	ref.	ref	
Grandfather smokes	Yes	744	136	18.3%	1.4 (1.1–1.7)	1.0 (0.8–1.2)	0.84
	No	5075	709	14.0%	ref.	ref..	
Grandmother smokes	Yes	152	44	28.9%	2.5 (1.7–3.5)	1.7 (1.1–2.4)	0.01
	No	5667	801	14.1%	ref.	ref.	
Sibling smokes	Yes	1103	310	28.1%	3.1 (2.6–3.6)	2.3 (1.9–2.7)	<0.001
	No	4716	535	11.3%	ref.	ref.	

OR: odds ratio, CI: confidence interval.

**Table 3:** Gender differences in smoking cessation.

Men							
**Variables**		***n***	**Current Smokers ** ***n*, %**		**Crude OR** **(95% CI)** **(*n* = 2142)**	**Adjusted OR** **(95% CI)** **(*n* = 2131)**	***p*-Value**
Age (years)	30–55	779	437	56.1%	2.8 (2.3–3.4)	2.4 (2.0–2.9)	<0.001
	56≤	1363	428	31.4%	ref.	ref.	
Body mass index	≥25.0	597	226	37.9%	0.9 (0.7–1.0)	0.8 (0.6–1.0)	0.02
	<25.0	1545	639	41.4%	ref.	ref.	
Father smokes	Yes	1311	619	47.2%	2.1 (1.8–2.6)	1.8 (1.4–2.1)	<0.001
	No	831	246	29.6%	ref.	ref.	
Mother smokes	Yes	131	69	52.7%	1.7 (1.2–2.4)	1.2 (0.8–1.7)	0.41
	No	2011	796	39.6%	ref.	ref.	
Grandfather smokes	Yes	240	134	55.8%	2.0 (1.5–2.7)	1.5 (1.1–2.0)	0.01
	No	1902	731	38.4%	ref.	ref.	
Grandmother smokes	Yes	57	29	50.9%	1.5 (0.9–2.6)	0.9 (0.5–1.6)	0.67
	No	2085	836	40.1%	ref.	ref.	
Sibling smokes	Yes	511	254	49.7%	1.6 (1.4–2.0)	1.3 (1.0–1.6)	0.03
	No	1631	611	37.5%	ref.	ref.	
Spouse smokes	Yes	168	93	55.4%	1.9 (1.4–2.7)	1.5 (1.1–2.1)	0.02
	No	1974	772	39.1%	ref.	ref.	
Cigarettes per day at initiation	≥21	351	137	39.0%	1.0 (0.9–1.2)	1.0 (0.9–1.2)	0.84
	11–20	1295	541	41.8%			
	1–10	496	187	37.7%	ref.	ref.	
Age at initiation (years)	<20	581	250	43.0%	1.2 (1.0–1.4)	0.8 (0.7–1.0)	0.08
	20≤	1561	615	39.4%	ref.	ref.	
Alcohol consumption (glasses)	≥3 (≥540 mL)	109	67	61.5%	1.3 (1.1–1.4)	1.2 (1.1–1.3)	0.003
	≥2–<3 (360–540 mL)	341	155	45.5%			
	≥1–<2 (180–360 mL)	789	306	38.8%			
	<1 (<180 mL)	903	337	37.3%	ref.	ref.	
**Women**							
**Variables**		***n***	**Current Smokers** ***n*, %**		**Crude OR** **(95% CI)** **(*n* = ** **845** **)**	**Adjusted OR** **(95% CI)** **(*n* = ** **834** **)**	***p-*** **V** **alue**
Age (years)	30–55	651	269	41.3%	0.9 (0.7–1.2)	0.8 (0.6–1.1)	0.19
	56≤	194	85	43.8%	ref.	ref.	
Body mass index	≥25.0	88	38	43.2%	1.0 (0.7–1.7)	1.1 (0.7–1.7)	0.81
	<25.0	757	316	41.7%	ref.	ref.	
Father smokes	Yes	569	256	45.0%	1.5 (1.1–2.0)	1.4 (1.0–1.9)	0.06
	No	276	98	35.5%	ref.	ref.	
Mother smokes	Yes	102	55	53.9%	1.7 (1.1–2.6)	1.6 (1.0–2.4)	0.04
	No	743	299	40.2%	ref.	ref.	
Grandfather smokes	Yes	136	60	44.1%	1.1 (0.8–1.6)	1.0 (0.7–1.5)	0.99
	No	709	294	41.5%	ref.	ref.	
Grandmother smokes	Yes	44	24	54.5%	1.7 (0.9–3.2)	1.5 (0.8–2.9)	0.20
	No	801	330	41.2%	ref.	ref.	
Sibling smokes	Yes	310	139	44.8%	1.2 (0.9–1.6)	1.0 (0.7–1.4)	0.91
	No	535	215	40.2%	ref.	ref.	
Spouse smokes	Yes	535	214	40.0%	0.8 (0.6–1.1)	0.8 (0.6–1.1)	0.22
	No	310	140	45.2%	ref.	ref.	
Cigarettes per day at initiation	≥21	32	13	40.6%	1.3 (1.1–1.7)	1.2 (0.9–1.6)	0.17
	11–20	251	125	49.8%			
	1–10	562	216	38.4%	ref.	ref.	
Age at initiation (years)	<20	206	90	43.7%	1.1 (0.8–1.5)	1.0 (0.7–1.4)	0.92
	20≤	639	264	41.3%	ref.	ref.	
Alcohol consumption (glasses)	≥3 (≥540 mL)	26	15	57.7%	1.4 (1.2–1.8)	1.4 (1.2–1.7)	0.001
	≥2–<3 (360–540 mL)	44	24	54.5%			
	≥1–<2 (180–360 mL)	149	79	53.0%			
	<1 (<180 mL)	626	236	37.7%	ref.	ref.	

OR: odds ratio, CI: confidence interval.

## Data Availability

This study was conducted by analyzing a dataset of the Nagahama Prospective Cohort for Comprehensive Human Bioscience (the Nagahama Study); the data are available upon request because we did not obtain consent from each participant for publication of individual data in the public domain. Data from the Nagahama study are available upon request to the Nagahama office (nagahama-office@genome.med.kyoto-u.ac.jp). We plan to make our data available at the National Bioscience Database Center after obtaining consent from the participants in this study.

## Supplementary Materials

The following are available online at https://www.mdpi.com/article/10.3390/ijerph19031511/s1, Questionnaire on specific health examination (Question relevant to the subject).

## References

[1] Schroeder S.A., Koh H.K. (2014). Tobacco Control 50 Years After the 1964 Surgeon General’s Report. JAMA.

[2] GBD 2015 Tobacco Collaborators (2017). Smoking prevalence and attributable disease burden in 195 countries and territories, 1990–2015: A systematic analysis from the Global Burden of Disease Study 2015. Lancet.

[3] World Health Organization (2011). Gender, Health, Tobacco and Equity. http://www.who.int/tobacco/publications/gender/gender_tobacco_2010.pdf?ua=1.

[4] World Health Organization (2017). WHO Report on the Global Tobacco Epidemic, 2017 Monitoring Tobacco Use and Prevention Policies.

[5] Organization for Economic Cooperation and Development Smoking among Adults Health at a Glance 2019; pp. 88–89.

[6] Smith P.H., Kasza K.A., Hyland A., Fong G.T., Borland R., Brady K., Carpenter M.J., Hartwell K., Cummings K.M., McKee S.A. (2015). Gender differences in medication use and cigarette smoking cessation: Results from the international tobacco control four country survey. Nicotine Tob. Res..

[7] Verplaetse T.L., Weinberger A.H., Smith P.H., Cosgrove K.P., Mineur Y.S., Picciotto M.R., Mazure C.M., McKee S.A. (2015). Targeting the noradrenergic system for gender-sensitive medication development for tobacco dependence. Nicotine Tob. Res..

[8] Fidler J., Ferguson S.G., Brown J., Stapleton J., West R. (2013). How does rate of smoking cessation vary by age, gender and social grade? Findings from a population survey in England. Addiction.

[9] Lam C.Y., Businelle M.S., Cofta-Woerpel L., McClure J.B., Cinciripini P.M., Wetter D.W. (2014). Positive smoking outcome expectancies mediate the relation between alcohol consumption and smoking urge among women during a quit attempt. Psychol. Addict. Behav..

[10] Jarvis M.J., Cohen J.E., Delnevo C.D., Giovino G.A. (2013). Dispelling myths about gender differences in smoking cessation: Population data from the USA, Canada and Britain. Tob. Control..

[11] Smith P.H., Bessette A.J., Weinberger A.H., Sheffer C.E., McKee S.A. (2016). Sex/gender differences in smoking cessation: A review. Prev. Med..

[12] Lee A., Lee K., Lee D., Ahn H., Lee H., Kim H., Lee J., Seo H. (2021). The Utilization of National Tobacco Cessation Services among Female Smokers and the Need for a Gender-Responsive Approach. Int. J. Env. Res. Public Health.

[13] Ozbay N., Shevorykin A., Smith P., Sheffer C.E. (2020). The association between gender roles and smoking initiation among women and adolescent girls. J. Gend. Stud..

[14] Leonardi-Bee J., Jere M.L., Britton J. (2011). Exposure to parental and sibling smoking and the risk of smoking uptake in childhood and adolescence: A systematic review and meta-analysis. Thorax.

[15] Melchior M., Chastang J.F., Mackinnon D., Galera C., Fombonne E. (2010). The intergenerational transmission of tobacco smoking—The role of parents’ long-term smoking trajectories. Drug Alcohol Depend..

[16] Vandewater E.A., Park S.E., Carey F.R., Wilkinson A.V. (2014). Intergenerational transfer of smoking across three generations and forty-five years. Nicotine Tob. Res..

[17] El-Amin S.E., Kinnunen J.M., Ollila H., Helminen M., Alves J., Lindfors P., Rimpelä A.H. (2016). Transmission of Smoking across Three Generations in Finland. Int. J. Environ. Res. Public Health.

[18] Setoh K., Terao C., Muro S., Kawaguchi T., Tabara Y., Takahashi M., Nakayama T., Kosugi S., Sekine A., Yamada R. (2015). Three missense variants of metabolic syndrome-related genes are associated with alpha-1 antitrypsin levels. Nat. Commun..

[19] Shigakokuho RESULT OF SPECIFIC MEDICAL CHECKUP. http://www.shigakokuho.or.jp/cmsfiles/contents/0000000/61/english2.pdf.

[20] Sun L., Tan L., Yang F., Luo Y., Li X., Deng H.W., Dvornyk V. (2012). Meta-analysis suggests that smoking is associated with an increased risk of early natural menopause. Menopause.

[21] World Health Organization (2021). WHO Report on the Global Tobacco Epidemic 2021: Addressing New and Emerging Products; Monitor Tobacco Use and Prevention Policies.

[22] Kandel D.B., Griesler P.C., Hu M.C. (2015). Intergenerational Patterns of Smoking and Nicotine Dependence Among US Adolescents. Am. J. Public Health.

[23] Brook J.S., Rubenstone E., Zhang C., Finch S.J., Brook D.W. (2013). The intergenerational transmission of smoking in adulthood: A 25-year study of maternal and offspring maladaptive attributes. Addict Behav..

[24] Kandel D.B., Wu P., Davies M. (1994). Maternal smoking during pregnancy and smoking by adolescent daughters. Am. J. Public Health Res..

[25] Munafo M.R., Johnstone E.C. (2008). Genes and cigarette smoking. Addiction.

[26] Lerman C., Shields P.G., Wileyto E.P., Audrain J., Pinto A., Hawk L., Krishnan S., Niaura R., Epstein L. (2002). Pharmacogenetic investigation of smoking cessation treatment. Pharmacogenetics.

[27] Malaiyandi V., Lerman C., Benowitz N.L., Jepson C., Patterson F., Tyndale R.F. (2006). Impact of CYP2A6 genotype on pretreatment smoking behaviour and nicotine levels from and usage of nicotine replacement therapy. Mol. Psychiatry.

[28] Lee A.M., Jepson C., Hoffmann E., Epstein L., Hawk L.W., Lerman C., Tyndale R.F. (2007). CYP2B6 genotype alters abstinence rates in a bupropion smoking cessation trial. Biol. Psychiatry.

[29] Tanner J., Prasad B., Claw K.G., Stapleton P., Chaudhry A., Schuetz E.G., Thummel K.E., Tyndal R.F. (2017). Predictors of Variation in CYP2A6 mRNA, Protein, and Enzyme Activity in a Human Liver Bank: Influence of Genetic and Nongenetic Factors. J. Pharmacol. Exp. Ther..

[30] World Health Organization (2019). WHO Report on the Global Tobacco Epidemic, 2019; Offer Help to Quit Tobacco Use.

[31] Stephens T., Pederson L.L., Koval J.J., Macnab J. (2001). Comprehensive tobacco control policies and the smoking behaviour of Canadian adults. Tob. Control..

[32] de Kleijn M.J.J., Farmer M.M., Booth M., Motala A., Smith A., Sherman S., Assendelft W.J.J., Shekelle P. (2015). Systematic review of school-based interventions to prevent smoking for girls. Syst. Rev..

[33] Thomas R.E., Baker P.R., Thomas B.C., Lorenzetti D.L. (2015). Family-based programmes for preventing smoking by children and adolescents. Cochrane Database Syst. Rev..

[34] Sherman S.J., Chassin L., Presson C., Seo D., Macy J.T. (2009). The Intergenerational Transmission of Implicit and Explicit Attitudes toward Smoking. J. Exp. Soc. Psychol..

