# Supplementary material for: Gender Differences in Smoking Initiation and Cessation Associated with the Intergenerational Transfer of Smoking across Three Generations: The Nagahama Study
Source: Int J Environ Res Public Health. 2022 Jan 28;19(3):1511. doi: 10.3390/ijerph19031511 (PMC8835433; doi:10.3390/ijerph19031511)
Supplement: Supplementary file 1 [file ijerph-19-01511-s001.zip › ijerph-1550471-supplementary.pdf]

Questionnaire on specific health examination (Question relevant to the subject)

Gender 1. Male 2. Female

- How old are you? ________________ years old

- Are you a current regular smoker?
  - A "current regular smoker" is a person who has smoked a total of 100 or more cigarettes or smoked for 6 months or longer and has been smoking for the last one month.

  1. Yes (Current) 2. Ever 3. Never

(If you answered 1. Yes (Current),)

- What is the number of cigarettes smoked per day (CPD) at the present?
  ________________ cigarettes

(If you answered 1. Yes (Current) or 2. Ever,)

- How long have or had you been smoking?
  ________________ Years
- When did you start smoking?
  ________________ years old
- What was the number of cigarettes smoked per day (CPD) at the time of smoking initiation? ________________ cigarettes

- Whether or not your family members smoke or smoked?
  - ✓ Mother 1. Yes 0. No

    If mother was chosen as a smoker, please check her smoking status during pregnancy. 1. Yes 0. No 2. unknown
  - ✓ Father 1. Yes 0. No
  - ✓ Grandmother 1. Yes 0. No
  - ✓ Grandfather 1. Yes 0. No
  - ✓ Sib-lings 1. Yes 0. No
  - ✓ Spouse 1. Yes 0. No

- How often do you drink alcohol (Sake, shochu [distilled spirits], beer, liquor, etc.)?

  1. Everyday　2. Sometimes　3.Rarely drink（cann’t drink）

- How much do you drink a day, in terms of glasses of refined Japanese Sake? (A glass [180mL] of refined Sake (rice wine) is equivalent to a medium bottle [500mL] of beer, 110mL of shochu (alcohol content 25 percent), a glass [double, 60mL] of whisky, and two glasses [240mL] of wine)

  1. <1 (less than 180mL)
  2. ≥1 and <2 (180-360mL)
  3. ≥2 and <3 (360-540mL)
  4. ≥3 (more than 540mL)
